# Simultaneous Derivative Spectrophotometric Analysis of Doxylamine Succinate, Pyridoxine Hydrochloride and Folic Acid in Combined Dosage Forms

**DOI:** 10.4103/0250-474X.44607

**Published:** 2008

**Authors:** A. Pathak, S. J. Rajput

**Affiliations:** Quality Assurance Laboratory, Pharmacy Department, Faculty of Technology and Engineering, The Maharaja Sayajirao University of Baroda, Vadodara-390 001, India

**Keywords:** Doxylamine succinate, pyridoxine hydrochloride, folic acid, derivative spectrophotometry

## Abstract

Two UV spectrophotometric methods have been developed, based on first derivative spectrophotometry for simultaneous estimation of doxylamine succinate, pyridoxine hydrochloride, and folic acid in tablet formulations. In method I, the concentrations of these drugs were determined by using linear regression equation. Method II is also based on first derivative spectrophotometry however simultaneous equations (Vierdot's method) were derived on derivative spectra. The first derivative amplitudes at 270.0, 332.8 and 309.2 nm were utilized for simultaneous estimation of these drugs respectively by both methods. In both the methods, linearity was obtained in the concentration range 2.5-50 μg/ml, 1-40 μg/ml and 1-30 μg/ml for doxylamine succinate, pyridoxine hydrochloride, and folic acid respectively. The developed methods show best results in terms of linearity, accuracy, precision, LOD, LOQ and ruggedness for standard laboratory mixtures of pure drugs and marketed formulations. The common excipients and additives did not interfere in their determinations.

Doxylamine succinate (DOX) is chemically N,N-dimethyl-2-[α-methyl-α-(2-pyridyl)benzyloxy] ethylamine hydrogen succinate. It is an antihistaminic with antimuscarinic and pronounced sedative effect^1^. It is official in USP^2^. Pyridoxine hydrochloride (PYR) is chemically 3-hydroxy-4,5-bis (hydroxymethyl)-2-picoline hydrochloride. It is a water soluble vitamin and involved principally in amino acid, carbohydrate and fat metabolism. It is required for the formation of haemoglobin^3^. It is official in IP^4^, BP^5^ and USP^6^. Folic acid (FA) chemically 4-(2-amino-4-hydroxypteridin-6-yl) methyl aminobenzoyl-l-glutamic acid, part of the vitamin B group (vitamin B9) is a water soluble vitamin. It is one of the most important coenzyme of the haemopoietic system that controls the generation of ferrohaeme^7^. Marketed tablet formulations of these agents play an important role in the treatment of persistent nausea and vomiting during pregnancy.

Several analytical methods have been reported for the determination of DOX or PYR or FA either alone or in combination with other drugs, using HPLC^8^,^9^, HPTLC^10^, UV 11–16, spectrofluorometric determination^17^,^18^, capillary electrophoresis^19^ and flow injection^20^. These methods have not been studied for use in the simultaneous determination of DOX, PYR, and FA. There is no published literature dealing with simultaneous quantification of DOX, PYR, and FA in bulk material and pharmaceutical preparations. Thus the present aim of the study is to develop simple, rapid and economical methods for the simultaneous estimation of these drugs in combined tablet dosage form.

Two spectrophotometric method have been developed for the simultaneous determination of doxylamine succinate (DOX), pyridoxine hydrochloride (PYR) and folic acid (FA) by using zero crossing first derivative methodology (method I) and applying simultaneous equation (Vierdot's method) on derivative spectra (method II). All materials and reagents were of analytical-reagent grade. Pure DOX, PYR and FA were procured as gift samples from Mercury Lab, Vadodara.

The derivative UV spectra of standard and test solutions were recorded in 1 cm quartz cells using a Shimadzu UV/Vis-1700 double beam UV/Vis spectrophotometer (Japan) with a fixed slit width of 2 nm. The zero order and first derivative absorption spectra were recorded over the wavelength range 220-400 nm against the solvent blank.

The standard stock solutions (0.1 mg/ml) of DOX, PYR and FA in 0.1 N NaOH were prepared. Further dilutions were made in 0.1 N NaOH to obtain concentrations ranging from 2.5-50 μg/ml for DOX, 1-40 μg/ml for PYR and 1-30 μg/ml for FA. The absorbance of resulting solutions was measured at 270.0, 332.8 and 309.2 nm for DOX, PYR and FA respectively and the calibration curves were plotted at these wavelengths.

The overlain zero order spectra of DOX, PYR and FA (fig. 1) showed that the absorption maxima of DOX PYR and FA lie in close proximity and at absorption maxima of one, another exhibits substantial absorbance. This clearly indicates the existence of spectral interference in estimation of DOX and PYR and FA. To overcome this, spectra of all three drugs were derivatised to first order between 220-400 nm with Δλ= 8 nm and scaling factor= 10. The overlain first derivative spectra of DOX, PYR and FA (fig. 2) reveal that PYR concentration is proportional to the first derivative signals at 332.8 nm (zero-crossing point for DOX and FA) and FA can be estimated at 309.2 nm (zero-crossing point for DOX and PYR). DOX was estimated from first derivative amplitude at 270.0 nm because here PYR and FA show amplitude of same magnitude with opposite signals resulting net contribution by both interferents as zero.

**Fig. 1:**
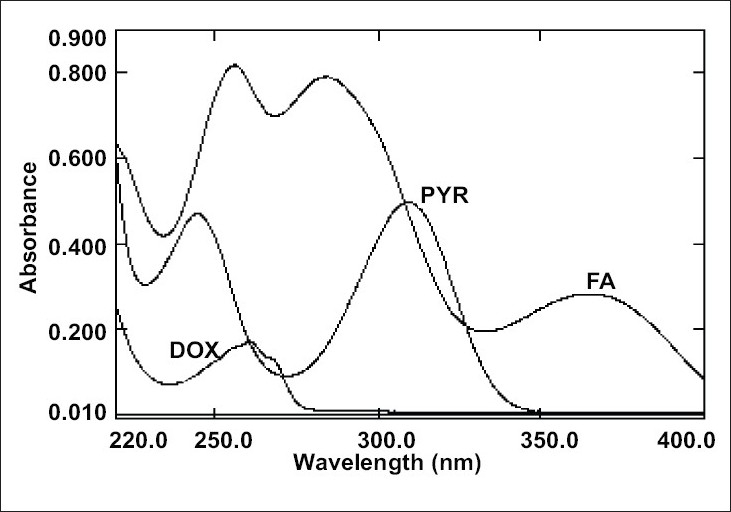
Overlain zero order spectra of DOX, PYR and FA. The spectra of the DOX, PYR and FA were taken for their 15 μg/ml solution.

**Fig. 2:**
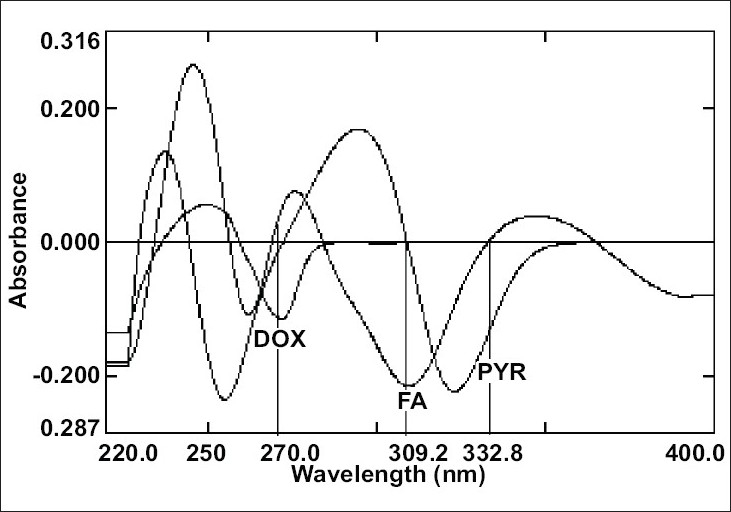
Overlain first derivative spectra of DOX, PYR and FA. The DOX, PYR and FA were estimated at the marked wavelengths 270.0, 309.2 and 332.8 nm respectively.

The linearity between first derivative amplitude and concentration of DOX, FA and PYR were examined at selected wavelengths, i.e. 270.0, 332.8 and 309.2 nm for DOX, PYR and FA respectively. Linearity was obtained in the range from 2.5-50 μg/ml for DOX, 1-40 μg/ml for PYR and 1-30 μg/ml for FA. The following linear equations obtained from standard plot were utilized for direct estimation of DOX, PYR and FA in the samples, C_DOX_ = 136.99×A_DOX_ -0.1506 (r = 0.9998) –(1), C_PYR_ = 108.69×A_PYR_ -0.1630 (r = 0.9998) –(2) and C_FA_ = 69.93×A_FA_ -0.133 (r = 0.9999) –(3), where, C_DOX_, C_PYR_ and C_FA_ are concentrations and A_DOX,_ A_PYR_ and A_FA_ are the absorbance of DOX, PYR and FA respectively in samples or mixed standards.

Three series of five mixed standards containing DOX, PYR and FA in the ratio (1:1:1), (4:4:1) and (10:10:1) in accordance with the ratio available in commercial formulations were prepared in 0.1 N NaOH and absorbance was measured at 270.0 nm, 332.8 nm and 309.2 nm for DOX, PYR and FA, respectively. By employing Eqns. (1), (2) and (3) in method I, the amplitudes contributed by DOX, PYR and FA to total amplitude at selected wavelengths were worked out.

Method II is also based on first derivative spectrophotometry, the wavelength selected for estimations of DOX, PYR and FA were same as described above. However, simultaneous equations (Vierdot's method) on derivative spectra were derived to overcome spectral interferences at selected wavelengths. First derivative absorptivity coefficients of individual drugs were determined at 270.0 nm, 332.8 nm and 309.2 nm. A set of three equations framed using these coefficient values is given below, C_DOX_ = 136.0296 DA_X_ –(4), C_PYR_ = 106.5270 DA_Z_ –(5) and C_FA_ = 68.1567 DA_Y_ –(6), where C_DOX_, C_PYR_ and C_FA_ are the concentration and DA_DOX_, DA_PYR_ and DA_FA_ are the absorbance of DOX, PYR and FA, respectively; DA_DOX_, DA_PYR_ and DA_FA_ are the first derivative amplitudes of mixture at 270.0, 332.8 and 309.2 nm. These Eqns were directly utilized for the simultaneous estimation of DOX, PYR and FA, respectively in standard laboratory mixture as well as the marketed formulations.

For the estimation of drugs in marketed tablet formulations, 10 tablets of each brand (Table 1) were separately weighed and powdered. The powder equivalent to 100 mg DOX for brand 1; 100 mg DOX for brand 2 and brand 3; and 100 mg DOX, 100 mg PYR and 100 mg FA for brand 4 was dissolved in 100 ml 0.1 N NaOH, ultrasonicated for 10 min, filtered, and the residue was washed with 0.1 N NaOH and the volume was adjusted to 100 ml with 0.1 N NaOH. Appropriate aliquots were then taken in such a way that the final concentrations in 10 ml volumetric flasks were within the range used for testing the drugs by the two methods. The amplitudes at 270.0 nm, 332.8 nm and 309.2 nm were recorded from the first derivative spectra. The concentration of each analyte was determined using the equations generated in both methods. Table 1 shows the results of all such analysis.

**TABLE 1:** RESULTS OF ANALYSIS OF COMMERCIAL FORMULATIONS

Formulations (Tablet)	Analyte	Labled mg/tablet	Method I		Method II	
				
			% mean±CI	SD	% mean±CI	SD
Brand 1	DOX	10	99.87±0.23	0.216	101.24±0.17	0.163
	PYR	10	99.46±0.20	0.186	99.00±0.15	0.141
	FA	1	98.88±0.59	0.301	99.34±0.31	0.296
Brand 2	DOX	10	100.21±0.12	0.111	99.69±0.09	0.089
	PYR	10	99.68±0.86	0.815	101.32±0.74	0.706
	FA	2.5	99.99±0.25	0.241	99.36±0.18	0.171
Brand 3	DOX	10	100.24±0.24	0.231	100.08±0.19	0.183
	PYR	10	99.82±0.58	0.555	103.01±0.57	0.541
	FA	2.5	100.84±0.18	0.174	99.83±0.10	0.091
Brand 4	DOX	10	102.01±0.09	0.071	101.64±0.07	0.068
	PYR	10	100.63±0.22	0.365	101.35±0.27	0.254
	FA	10	100.08±0.32	0.174	102.09±0.012	0.013

Method I is first derivative spectrophotometry method while Method II is the simultaneous equation method based on first derivative spectrophotometry. All the values indicated here are the mean values of five determinations. SD and CI denote the Standard deviation and Confidence Interval of analysis, which were calculated at 95% confidence level. Brand 1: Fedox (G Nine Formulations), Brand 2: PNV-D (Yash Pharma), Brand 3: Taurnate Plus (Taurus Lab) and Brand 4: Pyrinate (Helax Health Care).

The methods were validated according to International Conference on Harmonization guidelines for validation of analytical procedures^21^. Student's *t*-test and *F*-test were used to verify the validity of the methods. To study the recovery of DOX, PYR and FA, preanalyzed samples were taken to which known quantities of pure drugs (reference standards) were added in the concentration range 80% to 120%, but within the analytical range limit of the proposed methods. The added quantities of individual drugs were estimated by both developed methods and the results are calculated in terms of % recovery±confidence interval at 95% confidence level.

The intra-day and inter-day precisions of the proposed methods were determined by estimating the corresponding responses 3 times on the same day and 3 different days using 5 different concentrations of sample solution. From the absorbance obtained, concentration was calculated, and the results were expressed as% RSD.

The ruggedness of the UV method was determined by analysis of samples under a variety of conditions such as small changes in the normality of the NaOH solution (0.5-2.0 N) the method was also performed in different labs by using other UV instrument, and the percent relative standard deviation (%RSD) was calculated. The calibration curve was obtained with seven concentrations of the standard solution. The solutions were prepared in triplicate. The parameters Limit of Detection (LOD) and Limit of Quantitation (LOQ) were determined on the basis of response and slope of the regression equation.

The zero order absorption spectra of DOX, PYR and FA are represented in the fig. 1. The close overlap of the absorption spectra of DOX, PYR and FA prevents the correct use of zero-order absorption measurements for their simultaneous determination in ternary mixtures. On the other hand, the first-order spectrum (fig. 2) did not suffer any interference at the determination wavelength of DOX (270.0 nm), PYR (332.8 nm) and FA (309.2 nm), as expected.

Under the optimized conditions, the absorbance of the standard solutions of DOX, PYR and FA were measured at specified wavelengths. Linearity was obtained in the range from 2.5-50 μg/ml for DOX, 1-40 μg/ml for PYR and 1-30 μg/ml for FA. The coefficient of correlation as evaluated by least squares method in each case was > 0.99.

From this data, standard deviation (SD) of response and slope curve (S), it was possible to calculate the detection and quantitation limits. The LOD were 0.110 μg/ml, 0.193 μg/ml and 0.099 μg/ml and the value for LOQ were 0.369 μg/ml, 0.644 μg/ml and 0.331 μg/ml for DOX, PYR and FA, respectively. These low values indicated the good sensitivity of the methods proposed. The results of analysis of authentic samples and the average recoveries obtained in each instance were compared with theoretical value of 100 percent by means of students‘t’ test at 95 percent confidence level. The average percent recovery was found to be 100.59±0.13, 99.45±0.02 and 99.73±0.08 in method I and 99.80±0.62, 99.87±0.16 and 99.34±0.10 in method II for DOX, PYR and FA, respectively. The result of both methods lies within the prescribed limit of 98-102%, showing that both methods are free from interference from excipients. The results obtained from the intra-day and inter-day precision study were less than 2% (i.e., in the range of 0.590-1.070 and 0.845-1.234% for intra and inter day for proposed methods, respectively) indicating that the proposed methods were sufficiently precise for the analysis of drug. Both the methods were robust enough under different conditions as indicated by% RSD (i.e., in the range of 0.784-1.012% for proposed methods).

The applicability of the proposed methods for the determination of DOX, PYR and FA in commercial dosage forms was examined by analyzing marketed products. From Table 1, it is evident that there is good agreement between the amount estimated and those claimed by the manufacturers. Percent label claims are very close to 100, with low value of standard deviation. The proposed methods are sensitive, simple, and accurate and can be successfully applied for the quality control of pure DOX, PYR and FA in pharmaceutical dosage forms.
